# Feeding the Sick

**Published:** 1875-03

**Authors:** 


### FEEDING THE SICK.

"Stuff a cold and starve a fever" is a familiar saying, bearing the weight and authority of antiquity. But from an unfortunate obscurity in the language, the meaning of the sage to whom we are indebted for this bit of proverbial wisdom, is not always rightly understood. The sentence is generally supposed to express rules for the treatment of two special disorders. With this interpretation the individual suffering from a cold indulges in a diet palatable in quality and abundant in quantity. He stuffs the cold, or at least stuffs himself. We think it more probable that the wise man did not intend to advise, but rather to admonish,—as if he had said: "If you stuff a cold you will have a fever to starve." Adopting this interpretation, we recognize the wisdom of the proposition. Of course, the rule is not always verified. He who treats his cold to sumptuous fare does not always pay the penalty of a resulting fever. Many escape. But that they do so is no proof that they have adopted the most judicious treatment. They get well in spite of the treatment. A cold is, in medical language, *catarrhal fever*. Overloading the stomach and taxing the powers of the digestive organs to an unusual degree tends directly to increase that fever.

Much has been said of late in favor of the *dry cure* for colds. To take no liquids for two or three days is the course advised. While this treatment is suitable to most cases, we think that when the fever is well marked it would be better to abstain from solid food and take liquids freely. They increase the secretion of the skin, even to profuse perspiration, and allay febrile excitement. They may be of great service in diluting and washing away the contents of the stomach and bowels, which have become irritating and acrid.

Those very common disorders of digestion which are well enough designated by the convenient term *bilious*, will, in the great majority of cases, correct themselves if the organs implicated are allowed a season of partial repose. They have been over-worked. A few days of abstinence from food, or confinement to light and easily digested articles, will afford the stomach and liver an opportunity to recover their natural condition. That is, the patient will get well without the help of medicine. If *the people* would act upon this suggestion it would be all the better for them, and all the worse for the doctors.

Besides these disorders of the digestive organs which are corrected by a temporary light diet, we must mention the more serious disease known as dyspepsia. The name means *difficult digestion*, and is applicable whenever that difficulty exists, though it be the result of disease in the stomach or the liver, or in any other organ. It is not an easy task to lay down rules as to the food of a person suffering from chronic dyspepsia. To a great extent each patient is a law unto himself. Not all stomachs will welcome oatmeal mush, or wheaten grits, or graham bread. Before prescribing the diet of a dyspeptic we ask what articles of food best agree with him. The result of his own experience is worth more as a guide in his case than all the rules in all the books. There are dyspeptics who can, with impunity, eat a sufficient variety of articles to satisfy all the requirements of the system. But if they venture beyond certain limits they suffer the penalty inflicted by an offended stomach. It is folly for them to resort to medicines. The rational course is to follow the dictates of common sense, and eat those things which they have learned they can digest without difficulty. They are well enough if they will be content to let well enough alone.

Is it a good practice to "starve a fever"? The writer is not the oldest inhabitant in this city, but he remembers well when physicians would not give a patient burning with fever more than a teaspoonful of water to cool his parched tongue. The mouth, dry and crisp like parchment, the fiery skin, and the seething blood rushing through the veins, produced an intensity of suffering which, instead of being relieved, was made more terrible by the cruel mockery of water doled out drop by drop. It sometimes happened that, in spite of the earnest prohibitions of the doctor and the vigilant care of the nurse, the sufferer would manage to get hold of the pitcher, and from it drink one long, deep, sweet, delightful draught. In his agony he would drink though he knew it would be the death of him. But we never heard of a fever-patient killing himself in this way. After the stolen drink he very likely fell into a refreshing sleep, and awoke in a profuse perspiration—the fever gone. The doctor, if told the story of his patient's perverseness, was astonished that such a prodigious quantity of water did not kill him on the spot.

Now the fashion of medical practice has changed. The fever patient is allowed to drink as much as he pleases, with a reasonable care not to overload the stomach. Ice, in small pieces, allowed to dissolve in the mouth, allays the thirst and moistens the tongue without distending the stomach.

In the advanced stages of typhoid or other fever, as well as in some local inflammations like pneumonia, there is urgent need of drinks and food to supply the continued waste of the system, but the natural expressions of these wants through the sensations of thirst and hunger are absent. The patient remains for days, perhaps for weeks, in a state of unconsciousness more or less complete. His tongue is dry and cracked and brown, his lips parched, his skin husky, and, as the doctors used to say, the "secretions are all locked up." He may be, in fact, dying for the want of water, but he feels no thirst, nor any other desire, and will never ask for drink. Let a teaspoonful be placed between his lips, first arousing him from his lethargy, and he swallows it slowly, perhaps, by two or three efforts. His dull face shows, for a moment, an expression of satisfaction, and his friends say, "He seems to relish it." Though he has not the mental consciousness to realize a want, yet his entire physical frame is suffering from thirst, and it relishes the spoonful of water as the dry earth relishes the summer shower. The water must be given in this way at short intervals, perhaps every few minutes. In these cases there is the same want of food. If we wait until the patient's appetite returns before we feed him, he will probably starve to death. We must give him the most nutritious food, and in liquid form. He has not life enough to chew and swallow solid food, even if the stomach was able to dispose of it. The failing powers of the system must be restored by suitable food in small quantities, frequently repeated. Beef tea, egg-nog, wine-whey, clam soup, and similar articles, are preferable. Let the food be prepared in such a way as to be palatable; for the patient, with the first return of consciousness, will appreciate what is good, and be disgusted with what is inferior and poorly prepared.

It is admitted that a great many people have been killed by too much medicine. It is greatly to be feared that many, especially in the diseases referred to, have been starved. The most important rule in the treatment of fever may be thus negatively expressed: Do not kill the patient with medicine, and do not let him die for want of food.

During the stage of convalescence from dangerous diseases, great care must be exercised lest the patient make mistakes in the kind or the amount of his food. With returning health the appetite is often voracious, stronger than the judgment; and, if indulged imprudently, serious results may ensue. A general word of caution on this point is all that our present space allows.

It is our intention to give, in future numbers of this journal, more minute directions in relation to feeding the sick, and also to publish various recipes for the preparation of wholesome and palatable dishes for patients in various stages of sickness and convalescence, in order that our readers may be able to guard against under-feeding, as well as over-feeding, in such cases.

